# Effects of Formulation and Electrode Parameters on the Ignition Performance of Electrically Controlled Solid Propellant

**DOI:** 10.3390/ma19204297

**Published:** 2026-10-12

**Authors:** Rongyao Zhi, Zhe Zhang, Xiong Zhou, Yanan Wang, Gang Chen, Guodong Li, Yin Huang

**Affiliations:** 1 Hubei Key Laboratory of Theory and Application of Advanced Materials Mechanics, Wuhan University of Technology, Wuhan 430062, China; zhirongyao66@163.com (R.Z.); 13377880714@163.com (X.Z.); 13154729195@163.com (Y.W.); cg_chen@whut.edu.cn (G.C.); 2 National Key Laboratory of Aerospace Chemical Power, Hubei Institute of Aerospace Chemotechnology, Xiangyang 441003, China; zhangzhsvip@163.com

**Keywords:** electrically controlled solid propellant, mesh electrode, current density, thermal stress

## Abstract

This study investigates the effects of flame-retardant fillers, mesh electrode geometry, electrode material, propellant thickness, and applied voltage on the ignition and combustion performance of a perchlorate-based Electrically Controlled Solid Propellant (ECSP). Resistivity measurements show that phenolic resin (BS) increases resistivity by 82.8% (to 78.33 Ω·m), whereas decabromodiphenyl ether (BW) decreases it by 32.3% (to 29.04 Ω·m), relative to the base formulation (42.9 Ω·m). Finite element simulations reveal that current density concentrates at electrode–propellant contact points and decreases with increasing wire diameter and mesh number, whereas average current density exhibits a non-monotonic trend with mesh number. Thermal stress analysis indicates that pore size affects interfacial stress more significantly than wire diameter, and larger pores increase the risk of debonding. Ignition experiments demonstrate that asymmetric electrode configurations generate current density disparities that override polarity effects in determining the ignition sequence. The influence of electrode material follows the order Ti > SS > Cu and is independent of polarity. Ignition delay increases with propellant thickness, whereas thicknesses below 3 mm are prone to uncontrolled combustion. Under the present electrode configuration and test method, ignition proceeded very slowly at applied voltages below 60 V, whereas ignition occurred readily above 70 V. These findings provide practical guidance for electrode design and operational optimization in electrically controlled solid propulsion systems.

## 1. Introduction

Electrically Controlled Solid Propellants (ECSP) [1,2,3,4,5,6] are high-energy gel electrolytes that show considerable promise for future applications owing to their favorable flame insensitivity, high-temperature ablation resistance, and ability to combust upon electrical energization [7]. Based on the oxidizer type, ECSP can be broadly classified into ammonium nitrate-based, hydroxylammonium nitrate-based [8], and perchlorate-based formulations [9,10], each exhibiting distinct characteristics and application prospects.

The ignition process of ECSP is governed by multiple factors, including material composition [11,12,13,14,15], electrical parameters [16,17,18], and environmental conditions [19,20,21,22]. To date, extensive studies have been conducted on the effects of material composition and environmental conditions, covering oxidizer type and concentration, fuel type and stoichiometry, as well as temperature and pressure. Electrical parameters can be divided into supply voltage and current (including magnitude and waveform—direct or alternating current), whereas pulsed parameters include pulse width and frequency. In contrast, relatively less attention has been paid to electrode characteristics. Electrode parameters that influence the ECSP ignition sequence include polarity, material, and geometry. Adjusting these electrode parameters offers a means to control the ignition performance of ECSP [23,24,25,26].

According to the electrode arrangement, combustion-control electrodes for ECSP can be categorized into core-burning and end-burning configurations [27]. Owing to structural limitations of core-burning electrodes, the issue of electrical contact failure between the electrode and the propellant restricts their application to small-scale devices such as micro-thrusters, and prevents their direct scaling to larger rocket motors by increasing propellant grain length or diameter. In contrast, end-burning electrodes employ a specific electrode layout that enables axial end-surface combustion of the propellant, fundamentally circumventing the contact failure problem inherent in core-burning designs. This combustion mode imposes stringent requirements on electrical contact stability during the entire combustion process: effective contact between the propellant grain and the electrodes must be maintained throughout burning; otherwise, ignition failure or combustion interruption may occur, compromising the reliability of the propulsion system. Previous studies have achieved effective contact by driving electrode movement via elastic force [28], electromagnetic force [29], or gravity [21], thereby ensuring sustained and stable energy input and combustion stability. Current ECSP ignition experiments typically employ flat-plate electrodes. However, in practical applications, flat-plate electrodes tend to block gas flow passages, leading to combustion failure and other issues. Compared with flat-plate electrodes, mesh electrodes distribute current more uniformly over the propellant surface, which can increase current density and promote rapid formation of conductive pathways on the propellant surface. Mesh electrodes generally exhibit lower mechanical robustness; therefore, the electrode material must possess adequate mechanical properties to maintain structural integrity during ignition. Commonly used electrode materials for ECSP ignition include titanium (Ti), aluminium (Al), copper (Cu), and stainless steel (SS), which offer high mechanical strength and melting points, retain good mechanical performance at elevated temperatures, and remain structurally stable during the ECSP ignition process. Furthermore, by adjusting the geometric parameters of the mesh electrode, the current density distribution at the propellant interface can be modified, enabling directional control of the combustion zone and thereby enhancing the controllability of ECSP combustion. Mesh electrodes not only supply the electrical energy required for ECSP combustion but also facilitate the efficient venting of combustion gases, promoting stable combustion.

## 2. Materials and Methods

**Figure 1:**
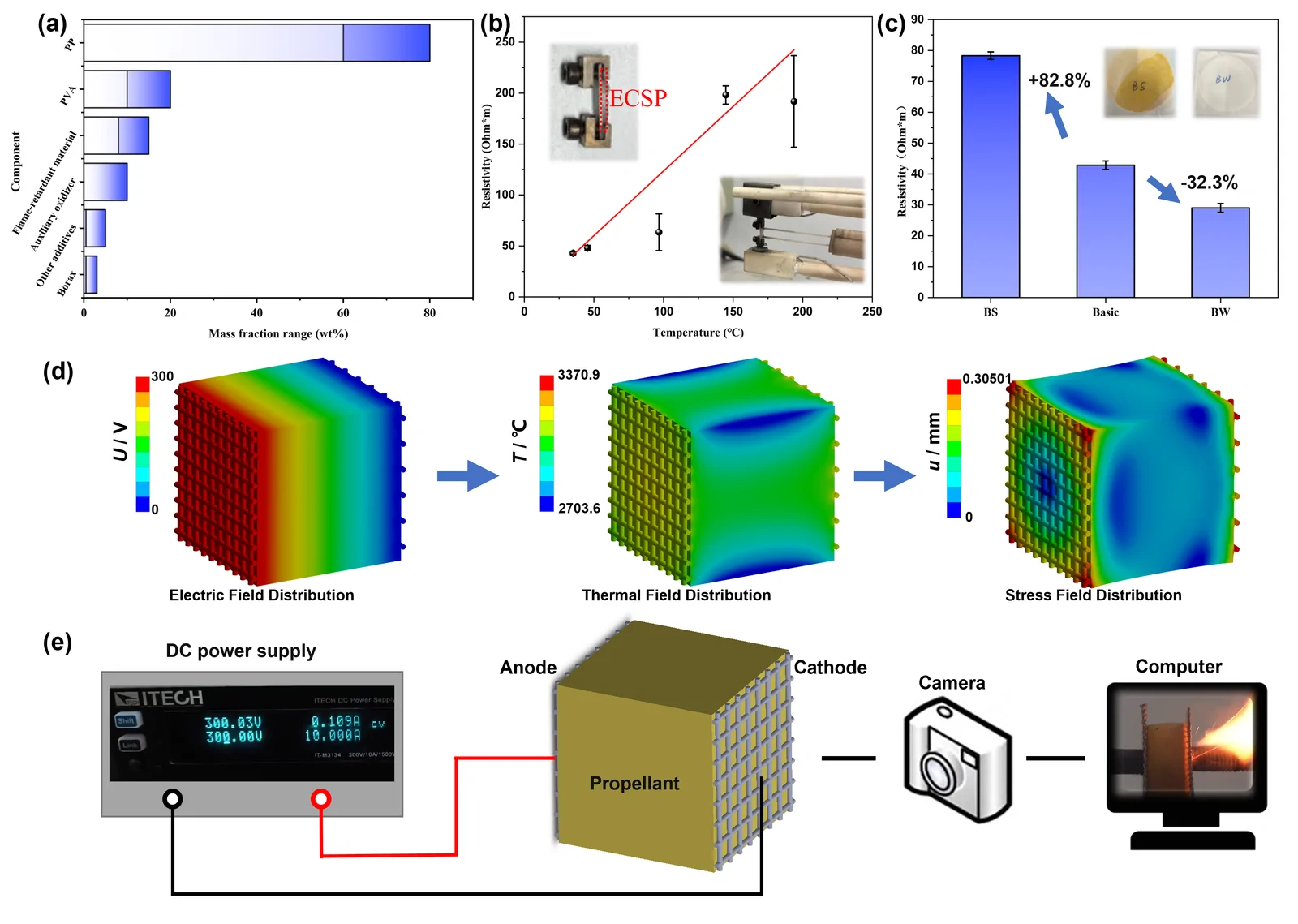
(**a**) Formulation and compositional content of the ECSP; (**b**) temperature-dependent resistivity of the unfilled ECSP measured over the range of 20–200 °C; (**c**) room-temperature resistivity of ECSP samples with different flame-retardant fillers (BS: phenolic resin; BW: decabromodiphenyl ether); (**d**) electro–thermal–mechanical coupled finite element simulation platform; (**e**) experimental setup for ECSP combustion testing.

The ECSP used in this study was synthesized by the Hubei Institute of Aerospace Chemical Technology. The primary raw materials included polyvinyl alcohol (PVA), a proprietary perchlorate-based oxidizer, a secondary oxidizer, ablation-resistant flame-retardant fillers, borax, and other additives. The base formulation is illustrated in Figure 1a. Based on literature survey and preliminary experimental results, phenolic resin and decabromodiphenyl ether were selected as flame-retardant fillers. The preparation procedure for the ECSP samples is illustrated in Figure S1. The samples were prepared by room-temperature mixing and elevated-temperature curing. According to the formulation, the secondary oxidizer was first added to the primary oxidizer and stirred until uniformly dispersed to obtain a mixed oxidizer solution. Functional additives were then added to the mixed oxidizer solution, and the mixture was stirred at room temperature to obtain a mixed solution. Subsequently, PVA, flame-retardant and ablation-resistant materials, crosslinking agent, and other components were added to the mixed solution, and the mixture was stirred under vacuum at room temperature to obtain a uniform, flowable propellant slurry. Finally, the propellant slurry was cast into a mold under vacuum and held at a constant temperature of 50 °C for 5–7 days for curing and forming.

Electrical conductivity was determined via the standard four-probe method using a CTA-3 instrument (Cryoall) under high-purity argon atmosphere, with resistivity calculated from the measured resistance. ECSP pellets (6.3 × 3.98 × 1 mm) were prepared and mounted on the test stage, and resistivity was measured from room temperature to 200 °C. Each data point was obtained from three repeated measurements on the same instrument, and the results are presented as the mean ± standard deviation in Figure 1b.

At room temperature, the average resistivity of the base-formulation ECSP was 42.9 Ω·m. With increasing temperature, the resistivity increased, which is attributed to decomposition and phase transitions; structural evolution at elevated temperatures also contributed to the observed variations.

In addition, two ECSP formulations containing different fillers were tested. Their resistivities remained relatively stable over multiple measurements at room temperature; therefore, all subsequent measurements for the filled samples were conducted at ambient temperature. Each measurement was repeated in three independent runs, and the final results are expressed as the mean ± standard deviation.

Samples denoted as BS contained phenolic resin as the flame-retardant filler, while BW samples contained decabromodiphenyl ether. The choice of filler exerted a marked influence on resistivity. The average resistivity of the BS formulation was 78.33 Ω·m, representing an 82.8% increase compared with the base formulation, whereas the BW formulation exhibited an average resistivity of 29.04 Ω·m, corresponding to a 32.3% decrease relative to the base formulation (Figure 1c). These results indicate that different flame-retardant fillers substantially modulate the resistivity of ECSP, which in turn may affect its ignition performance.

To investigate the influence of mesh electrode geometric parameters on the current density distribution across the ECSP end face, a finite element electrical conduction model was established using ANSYS 2021 R1 software. The simulation was solved based on the current conservation equation under quasi-static electric field conditions. After obtaining the potential distribution by solving this equation, the current density vector at each location was derived from the differential form of Ohm’s law. The ECSP and electrodes were arranged axially (Figure 1d). The conductivity at the contact interface was set to 10^3^ S·m^−2^; this value was calculated from the equivalent resistance between the metal and ECSP. In the simulation, Ti was selected as the electrode material, with an electrical resistivity of 4.8 × 10^−7^ Ω·m and a thermal conductivity of 16.4 W·m^−1^·K^−1^. The ECSP material parameters were experimentally determined. For the boundary conditions, the required ignition voltage was applied to the anode, and the cathode was grounded at 0 V.

To further explore the effect of mesh electrode structure on interfacial stability between the electrode and propellant during ignition, a coupled electro–thermal–mechanical finite element model was developed on the basis of current density analysis. This model was used to simulate Joule heating distribution and thermal stress evolution at the interface immediately upon power application, as shown in Figure 1d. The simulation conditions were set to replicate the ignition experiments as closely as possible, including applied voltage, convective heat transfer, and fixed support constraints. Convective heat transfer with air was assumed around the propellant, and the mechanical constraint was defined as a fixed support at the center of the burning surface. The material properties are listed in Table 1.

The computational model was discretized using a hybrid structured-unstructured mesh scheme. The electrode regions were meshed with unstructured elements at an element size of 0.2 mm, whereas the ECSP region was meshed with a structured grid using a base element size of 0.5 mm. To adequately resolve the current density, temperature gradients, and interfacial contact conductance effects at the electrode-ECSP interface, local mesh refinement was applied to the electrode–propellant interface, where the element size was controlled at 0.1 mm. A mesh independence study was performed. The relative deviation of the calculated results was less than 5%, indicating that the mesh resolution satisfied the computational requirements. This mesh scheme was therefore adopted for the subsequent simulations.

A combustion diagnostic system was developed to evaluate the ignition and combustion performance of ECSP under electrically energized conditions. The effect of current density on the ignition performance of ECSP was verified by examining the ignition position. The system consisted of a DC power supply, a combustion test stand, and a camera, as illustrated in Figure 1e.

Ignition experiments were conducted on ECSP samples. The DC power supply was operated in constant-voltage mode, and the sample was clamped between electrodes before power was applied to initiate the experiment. The combustion process was simultaneously recorded by the camera, with images captured at the moment of ignition and other relevant data recorded during the test. The ignition delay time is defined as the interval from the instant the voltage is applied to the first observation of initial ignition at the interface. The complete ignition time is defined as the interval from voltage application to the moment when the burning surface has spread across the entire electrode-ECSP contact interface. The re-ignition delay time is defined as the interval from the reapplication of the same excitation voltage, after extinction of the initial ignition, to the recurrence of initial ignition.

## 3. Results

### 3.1. Ignition Process and Mechanism of ECSP with Asymmetric Electrode Configuration

**Figure 2:**
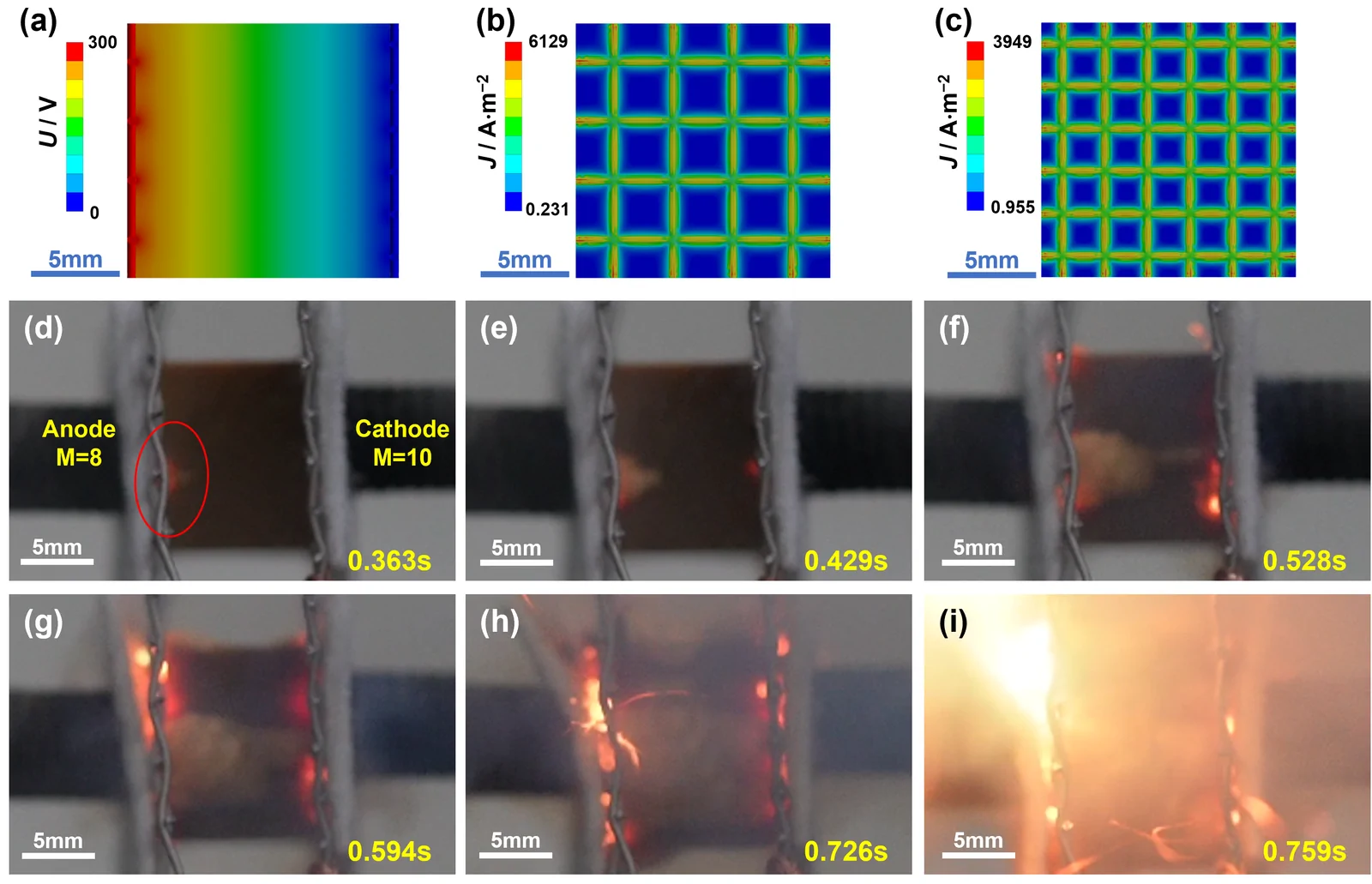
(**a**) Electric potential distribution of the electrode-ECSP assembly (**left**: anode; **right**: cathode); (**b**) current density distribution at the ECSP/8-mesh anode interface; (**c**) current density distribution at the ECSP/10-mesh cathode interface; (**d**–**i**) experimental images of the ECSP ignition process recorded at different times after power application (300 V, Ti mesh electrodes, 8-mesh anode, 10-mesh cathode): (**d**) at 0.363 s, onset of pyrolysis/liquefaction at the anode side.The position marked with a red circle is the initial ignition point.; (**e**–**g**) progressive expansion of the pyrolysis zone; (**h**) pyrolysis/liquefaction zone bridges the two electrodes, causing a sharp drop in overall resistance; (**i**) full combustion achieved. M denotes mesh number.

Ignition tests on ECSP have identified several factors that affect combustion performance, including electrode polarity, electrode material, electrode geometry, applied voltage, and propellant thickness. In the present study, cubic ECSP specimens with a side length of 10 mm were ignited using Ti mesh electrodes (wire diameter 0.5 mm, anode mesh number 8, cathode mesh number 10), and the ignition process was systematically analyzed. Figure 2a shows the electric potential distribution of the electrode-ECSP assembly, with the anode on the left and the cathode on the right. Finite element simulations were performed to obtain the current density distribution on the ECSP combustion end face, and the results are presented in Figure 2b,c. Specifically, Figure 2b displays the current density distribution at the interface between ECSP and the 8-mesh electrode, while Figure 2c shows that for the 10-mesh electrode interface.

When a voltage of 300 V was applied between the two electrodes, the 8-mesh anode, with its coarser grid (lower mesh number), had substantially fewer contact points with the ECSP than the 10-mesh cathode. Under the same total current, the current converges at discrete micro-contact spots, inducing a current constriction effect. Consequently, the local current density at these micro-contact spots is considerably higher than the average current density calculated from the apparent contact area. A reduction in the actual contact area decreases the ionic and electronic conduction pathways across the electrode–propellant interface, thereby increasing the interfacial contact resistance. Under a given applied voltage, a higher local current density is associated with a higher contact resistance, which increases the Joule heating power at the micro-contact regions and thus promotes interfacial heating. Accordingly, the initial peak in Joule heating tends to be preferentially concentrated on the anode side. Defining the instant of power application as 0 s, the Joule heat generated by the current ignited the contact points between the ECSP and the 8-mesh electrode, forming a liquefied and decomposed zone (Figure 2d). As the current increased, this zone gradually expanded toward the cathode, as shown in Figure 2e–g. Eventually, as depicted in Figure 2h, when the liquefied decomposition zone physically bridged the two electrodes, the overall resistance of the ECSP dropped abruptly, leading to a sharp surge in circuit current and a consequent burst of volumetric Joule heating (Figure 2i).

It is noteworthy that when the current densities at the anode and cathode are equal, ECSP exhibits a cathode-biased ignition characteristic, as reported in the literature. However, in the present configuration, the pronounced current density disparity between the 8-mesh and 10-mesh electrodes exerted a stronger influence on the ignition sequence than did electrode polarity.

### 3.2. Control of Current Density at the ECSP Burning Surface by Mesh Electrode Structural Parameters

**Figure 3:**
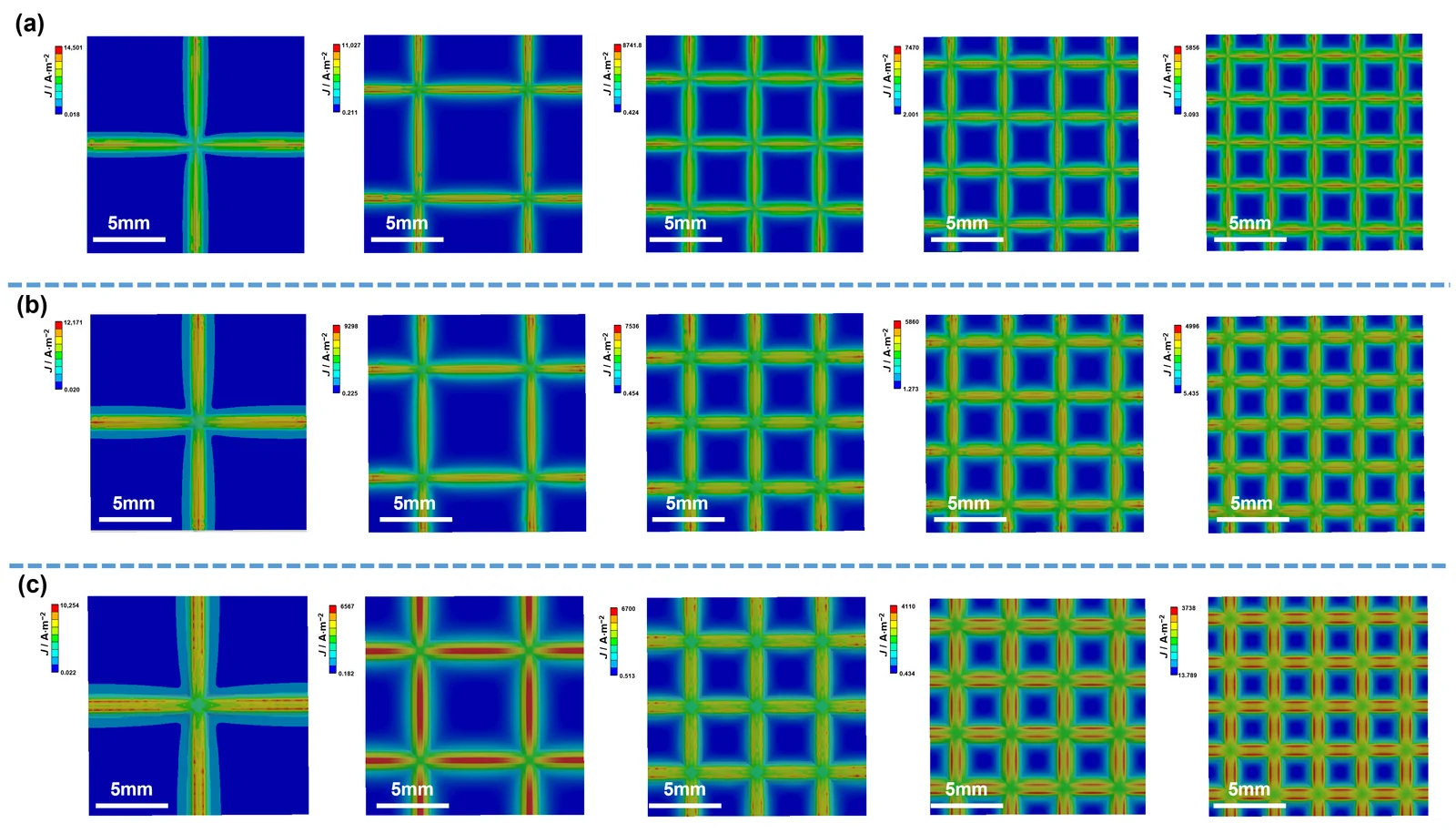
Contour plots of current density distribution on the ECSP combustion end face under different wire diameters and mesh numbers (300 V, Ti mesh electrodes). (**a**) Wire diameter 0.3 mm: current density distribution on the ECSP end face at varying mesh numbers; color scale from blue to red denotes low to high current density (unit: A/m^2^). Current density is concentrated at the contact points between the mesh electrode and the ECSP. (**b**) Wire diameter 0.5 mm. (**c**) Wire diameter 1 mm.

Current density distribution is a key factor governing the ignition performance of ECSP. To elucidate the effects of wire diameter and mesh number, mesh electrode models with wire diameters of 0.3 mm, 0.5 mm, and 1 mm were constructed. The mesh number was controlled by adjusting the number of metal wires in contact with the ECSP within a fixed area. By analyzing the current density distribution on the ECSP combustion end face, the preferential ignition location could be predicted, thereby revealing the influence of wire diameter and mesh number on combustion behavior. Detailed current density distributions on the ECSP end face for each electrode configuration are shown in Figure 3.

**Figure 4:**
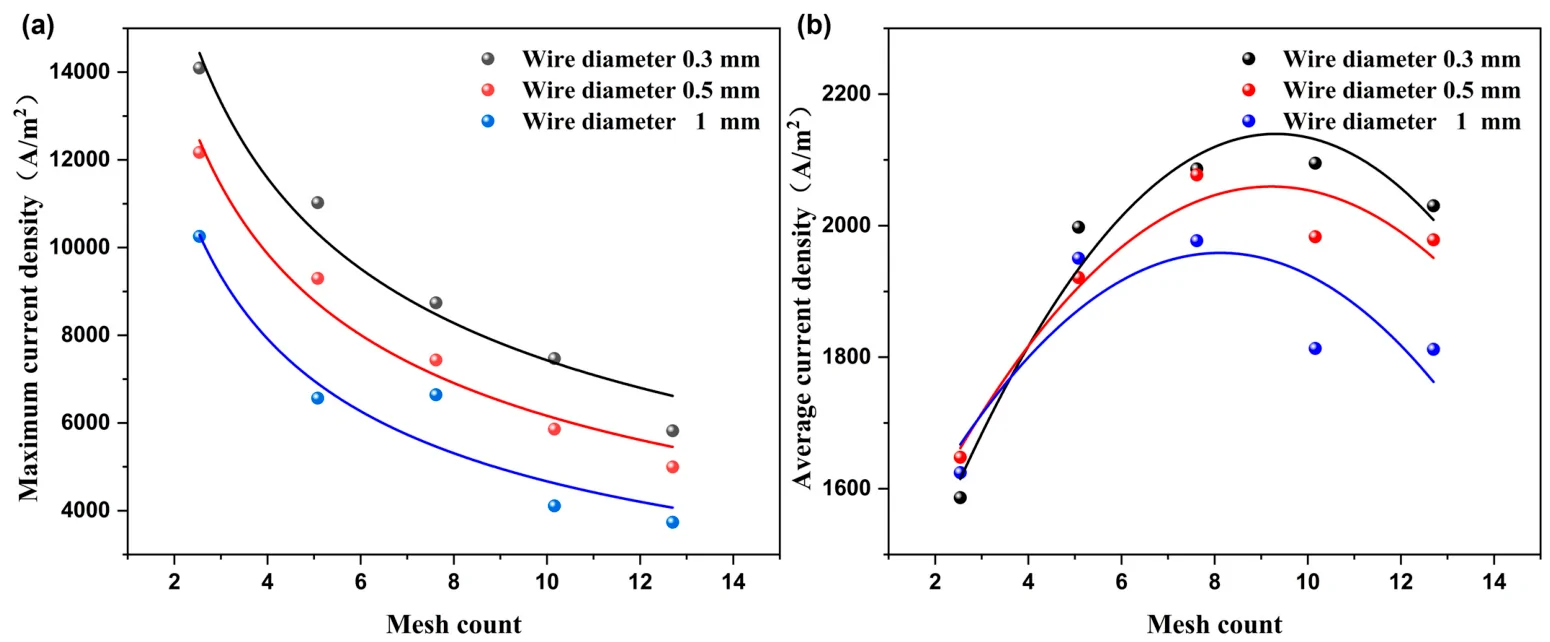
Simulated current density on the ECSP surface using SS mesh electrodes with different wire diameters and mesh numbers at 300 V. (**a**) Maximum current density as a function of wire diameter and mesh number; (**b**) average current density as a function of wire diameter and mesh number.

As shown in Figure 4a, the maximum current density at the ECSP-electrode interface decreased with increasing wire diameter and mesh number. Specifically, the interface current density for the 0.3 mm wire diameter electrode was 15.8% higher than that for the 0.5 mm wire diameter electrode. However, previous studies have indicated that reduced contact area between the electrode and ECSP leads to lower combustion efficiency, because regions not in direct contact with the electrode undergo neither pyrolysis nor electrolysis and thus cannot be ignited. This phenomenon is well reflected in the average current density on the ECSP combustion end face. As shown in Figure 4b, the average current density exhibited a non-monotonic trend, first increasing and then decreasing with increasing mesh number. When the mesh number is approximately 10-mesh, the average current density on the end face of ECSP reaches its maximum. Meanwhile, the current-density distribution on the ECSP end face is relatively uniform, which theoretically guarantees good ignition uniformity of ECSP.

### 3.3. Multi-Physical Field Analysis of the Electrode–Propellant Interface

When current passes through the electrodes and propellant, electrical resistance converts electrical energy into thermal energy, generating an intense and non-uniform temperature field. This temperature field, in combination with material thermal expansion characteristics and structural constraints, ultimately induces thermal stress at the interface. Mesh electrodes can substantially reduce ignition delay time compared with conventional plate electrodes. This is primarily because mesh electrodes produce more concentrated current density at the electrode–propellant contact points, resulting in stronger local Ohmic heating. However, this also gives rise to larger thermal stresses; excessive stress may cause contact failure at the electrode–propellant interface, adversely affecting the stable and reliable ignition of ECSP.

**Figure 5:**
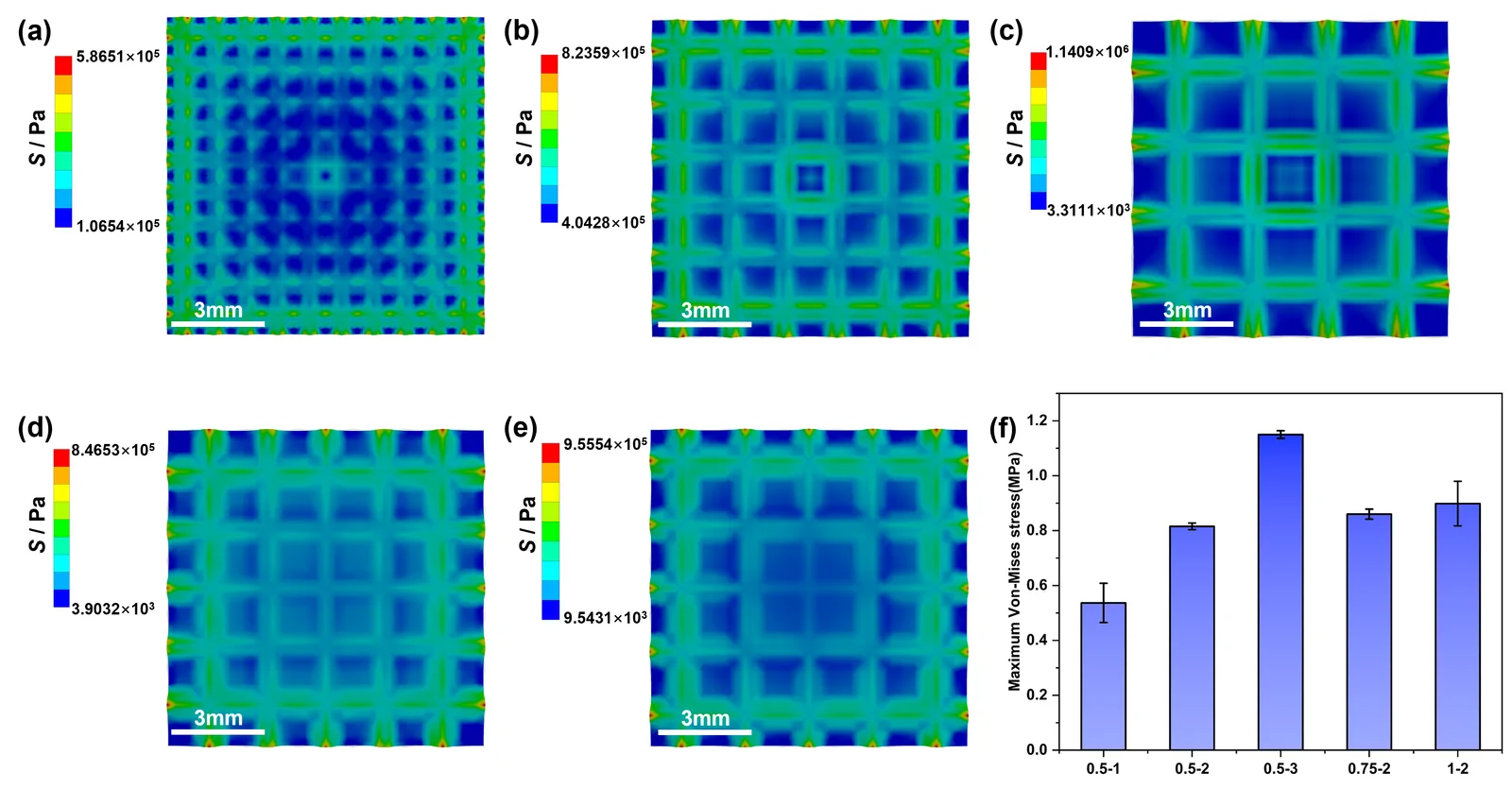
Thermal stress generated on the ECSP burning surface during power application under 300 V for different mesh electrode dimension combinations. (**a**) Wire diameter 0.5 mm, pore size 1 mm; (**b**) wire diameter 0.5 mm, pore size 2 mm; (**c**) wire diameter 0.5 mm, pore size 3 mm; (**d**) wire diameter 0.75 mm, pore size 2 mm; (**e**) wire diameter 1 mm, pore size 2 mm; (**f**) thermal stress on the ECSP combustion end face under the five electrode configurations.

In this work, five types of mesh electrodes with different wire diameters and pore sizes (wire diameter 0.5 mm with pore sizes 1, 2, and 3 mm; wire diameter 0.75 mm with pore size 2 mm; wire diameter 1 mm with pore size 2 mm) were designed to systematically investigate the respective effects of wire diameter and pore size on thermal stress distribution at the electrode–propellant interface. Cross-simulations using these five mesh electrode configurations were performed to obtain the equivalent thermal stress distribution on the ECSP burning surface in contact with each electrode. For each electrode configuration, the maximum equivalent stress on the ECSP end face at the electrode contact locations was extracted; these values were then de-meaned and their variance calculated to assess the influence of electrode parameters on the maximum thermal stress on the ECSP burning surface. The maximum equivalent thermal stress on the ECSP end face for each electrode configuration is shown in Figure 5. As the mesh electrode pore size increased, the maximum current density on the ECSP end face increased, and the resulting local hot spots became more intense, leading to higher local stress values on the combustion surface and greater deformation of the ECSP, which increased the likelihood of debonding and other interfacial failure issues. In contrast, variations in wire diameter had a relatively minor effect on this stress, with wire diameter primarily affecting the mechanical properties of the electrode itself. Therefore, for a wire diameter of 0.5 mm, adopting a mesh electrode with an appropriate pore size can ensure sufficient current density on the ECSP burning surface while maintaining relatively small thermal deformation.

**Figure 6:**
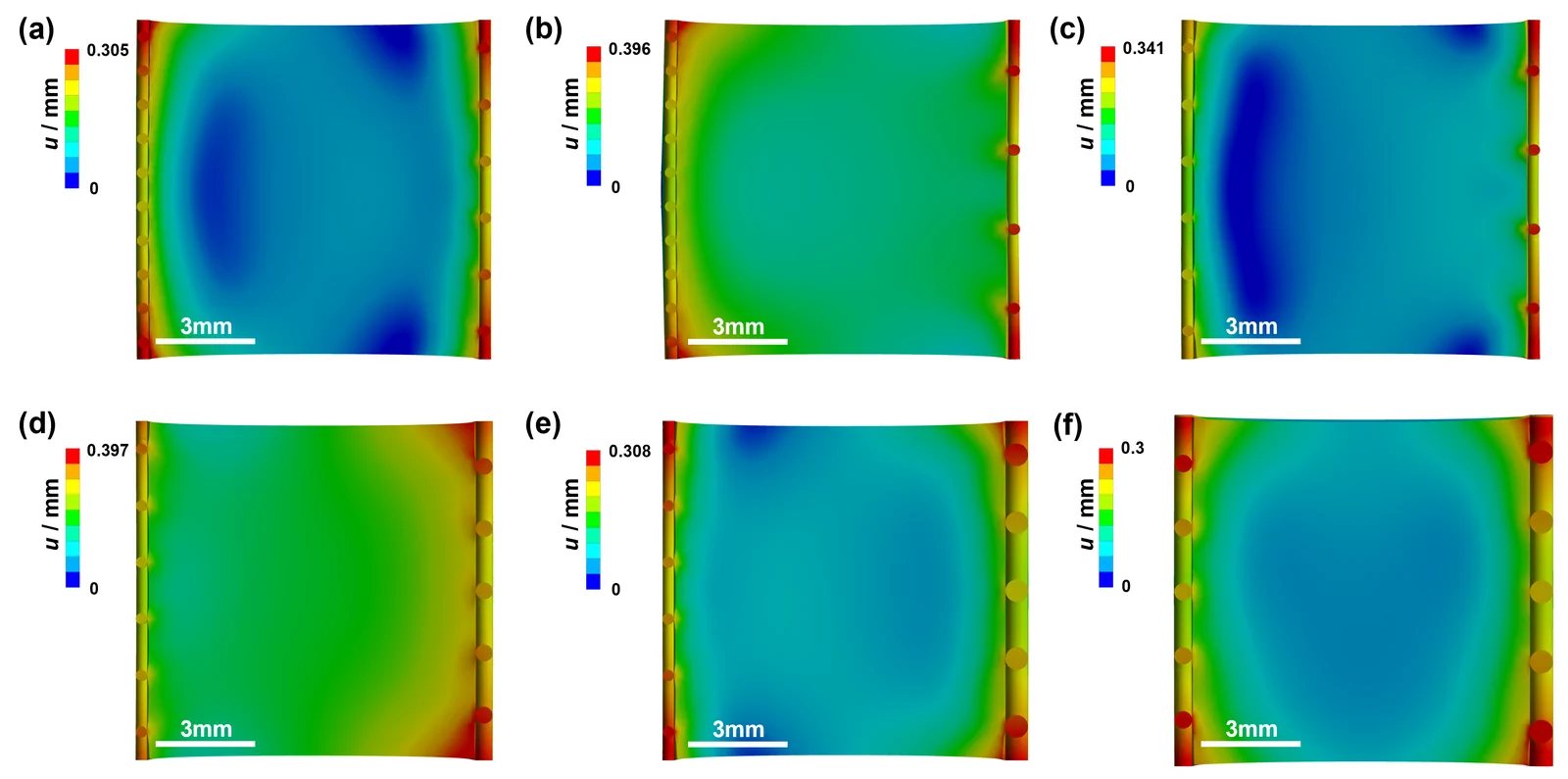
Thermal deformation of the electrode and ECSP during power application under 300 V for different mesh electrode dimension combinations. (**a**) Wire diameter 0.5 mm, pore sizes 1 and 2; (**b**) wire diameter 0.5 mm, pore sizes 1 and 3; (**c**) wire diameter 0.5 mm, pore sizes 2 and 3; (**d**) wire diameters 0.5 and 0.75 mm, pore size 2; (**e**) wire diameters 0.5 and 1 mm, pore size 2; (**f**) wire diameters 0.75 and 1 mm, pore size 2.

Figure 6 illustrates the deformation induced by Joule heating during the cross-simulation ignition tests of the five electrode configurations. Figure 6a, Figure 6b and Figure 6c show the cross-simulations for wire diameter 0.5 mm with pore sizes 1, 2, and 3 mm, respectively, examining the effect of pore size on the electrode–propellant interface. Figure 6d, Figure 6e and Figure 6f present the cross-simulations for pore size 2 mm with wire diameters 0.5, 0.75, and 1 mm, respectively, examining the effect of wire diameter on the interface. Compared with the other configurations, the mesh electrode with a wire diameter of 0.5 mm and pore size of 2 mm exhibited less deformation.

It can be clearly observed that the intersections of the electrode mesh wires are locations of maximum deformation, while the peripheral regions of the electrode are prone to warping. This observation explains why, in the ignition experiments, ignition preferentially occurred at the central region of the cross-section: this region contains more wire intersections and tighter point contacts, resulting in greater heat flux upon power application and thus a higher likelihood of ignition.

Greater differences in mesh electrode configuration also lead to localized hot spots in the overall structure, generating larger local thermal stresses and deformations, which are detrimental to stable ECSP ignition. Thermal deformation of the ECSP end face in contact with mesh electrodes of different dimensions is shown in Figures S2 and S3. Figures S2a and S2b correspond to the axial displacements of ECSP at the contact interfaces with mesh electrodes having a wire diameter of 0.5 mm and aperture sizes of 1 and 2, respectively, as shown in Figure 6a. For the left end face, the displacement is opposite to the axial direction and is therefore assigned a negative value. For the right end face, the displacement is opposite to the axial direction and is therefore assigned a positive value. The maximum axial displacement occurs at the contact position for the mesh electrode with a wire diameter of 0.5 mm and an aperture size of 3 mm, reaching approximately 0.22 mm. Increasing the wire diameter and decreasing the aperture size are beneficial for reducing the thermal stress generated at the ECSP end face, thereby decreasing the deformation. However, the aperture size has a greater influence than the wire diameter. In addition, an excessively large wire diameter reduces the current density at the ECSP end face and increases the material consumption of the device. Considering both the current density and the Joule heating-induced deformation of ECSP, the mesh electrode with a wire diameter of 0.5 mm and an aperture size of 2 mm provides better overall performance. This finding agrees with the simulation results for 10-mesh mesh electrodes, which yield the maximum average current density when investigating how structural parameters of mesh electrodes affect current density.

### 3.4. Effect of Mesh Electrode Material on ECSP Combustion Performance

To investigate the effect of mesh electrode material on the combustion performance of ECSP, ignition tests were conducted on cubic ECSP specimens with a side length of 10 mm. Ti, Cu, and SS mesh electrodes, all with a wire diameter of 0.3 mm and mesh number of 20, were used under a DC voltage of 300 V. In the setup, the left electrode was connected to the positive terminal and the right electrode to the negative terminal.

**Figure 7:**
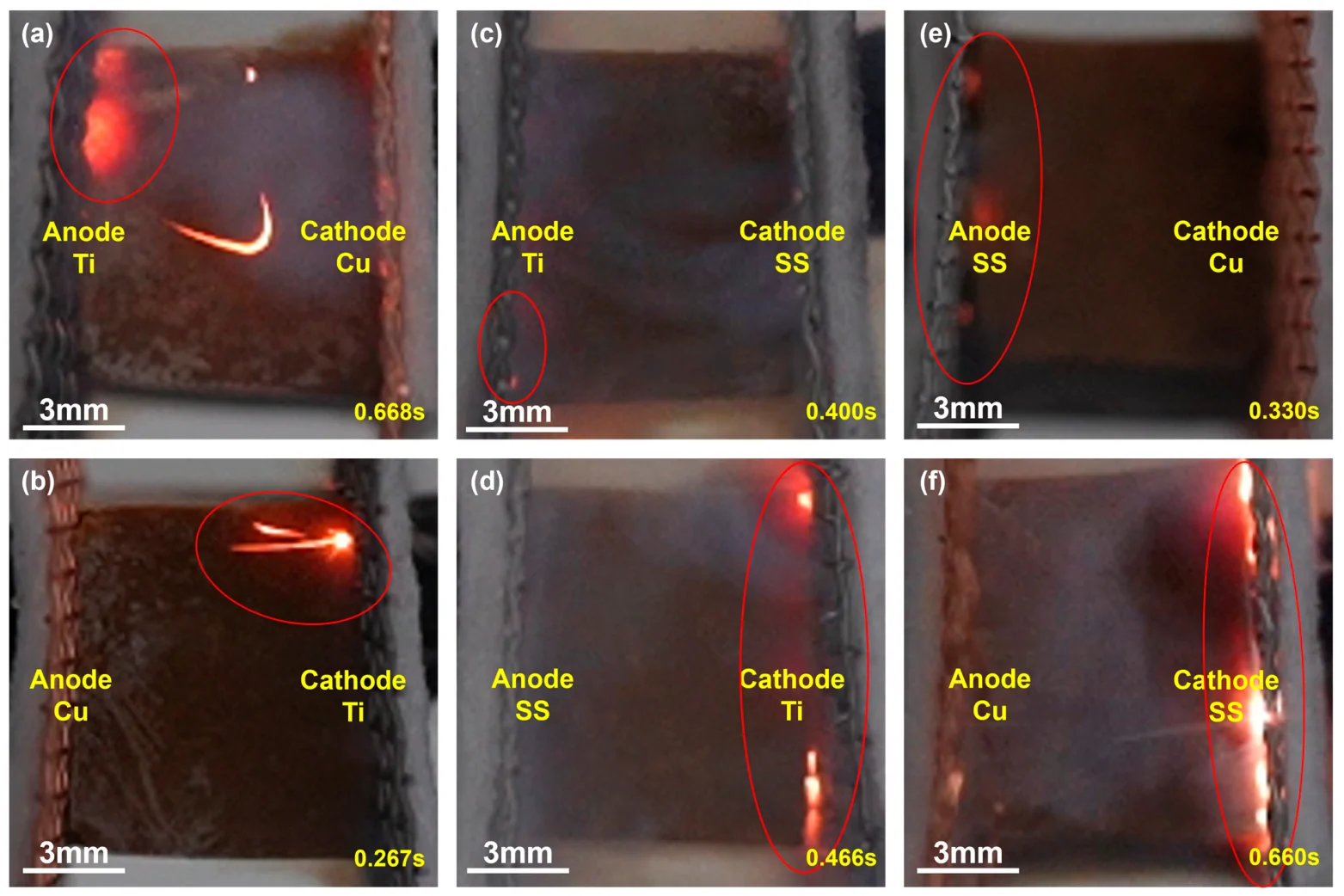
Ignition-instant images of ECSP with different electrode material combinations (300 V, wire diameter 0.3 mm, 20 mesh). (**a**) Anode Ti, cathode Cu; (**b**) anode Cu, cathode Ti; (**c**) anode Ti, cathode SS; (**d**) anode SS, cathode Ti; (**e**) anode SS, cathode Cu; (**f**) anode Cu, cathode SS. The position marked with a red circle is the initial ignition point.

Figure 7 shows the ignition-instant images of ECSP using Ti, Cu, and SS mesh electrodes. Specifically, Figure 7a corresponds to the configuration with Ti as the anode and Cu as the cathode; ignition was observed to initiate preferentially at the Ti electrode. To exclude any polarity-induced effects, the electrode polarity was reversed and the experiment was repeated. As shown in Figure 7b, ignition still occurred preferentially at the Ti electrode. Results for other electrode material combinations are presented in Figure 7c–f.

The experimental results indicate that, regardless of electrode polarity, the influence of electrode material on the ignition sequence follows the order: Ti > SS > Cu. Moreover, the effect of electrode material on the ECSP ignition sequence was found to be more pronounced than that of electrode polarity. This finding is consistent with the conclusions of Li et al. regarding the effect of electrode material on the ignition priority of ECSP.

Ti has the lowest electrical conductivity and the lowest thermal conductivity among the materials considered. Consequently, Joule heat generated at the interface is not readily dissipated through the electrode and tends to accumulate at the ECSP burning surface, facilitating hot-spot formation. Ti is a highly reactive metal and readily forms a thin, dense, strongly adherent TiO_2_ oxide film spontaneously. This oxide layer is thin and provides a moderate charge-transfer barrier. SS exhibits intermediate electrical conductivity and a higher thermal conductivity than Ti, allowing faster heat dissipation. In addition, Cr_2_O_3_ itself has a higher charge-transfer barrier, resulting in a larger intrinsic interfacial impedance. Cu has the highest bulk electrical conductivity, but its thermal conductivity is excessively high, so localized Joule heat at the interface dissipates rapidly, making interfacial hot spots difficult to sustain. Moreover, Cu oxides (CuO/Cu_2_O) have loose structures and poor adhesion and thus do not readily form stable hot spots on the ECSP burning surface. The bulk electrical conductivity of the metal is not the dominant factor; rather, thermal conductivity plays a key role in regulating interfacial heat retention and charge-transfer capability.

### 3.5. Effect of Propellant Thickness on the Combustion Performance of ECSP

**Figure 8:**
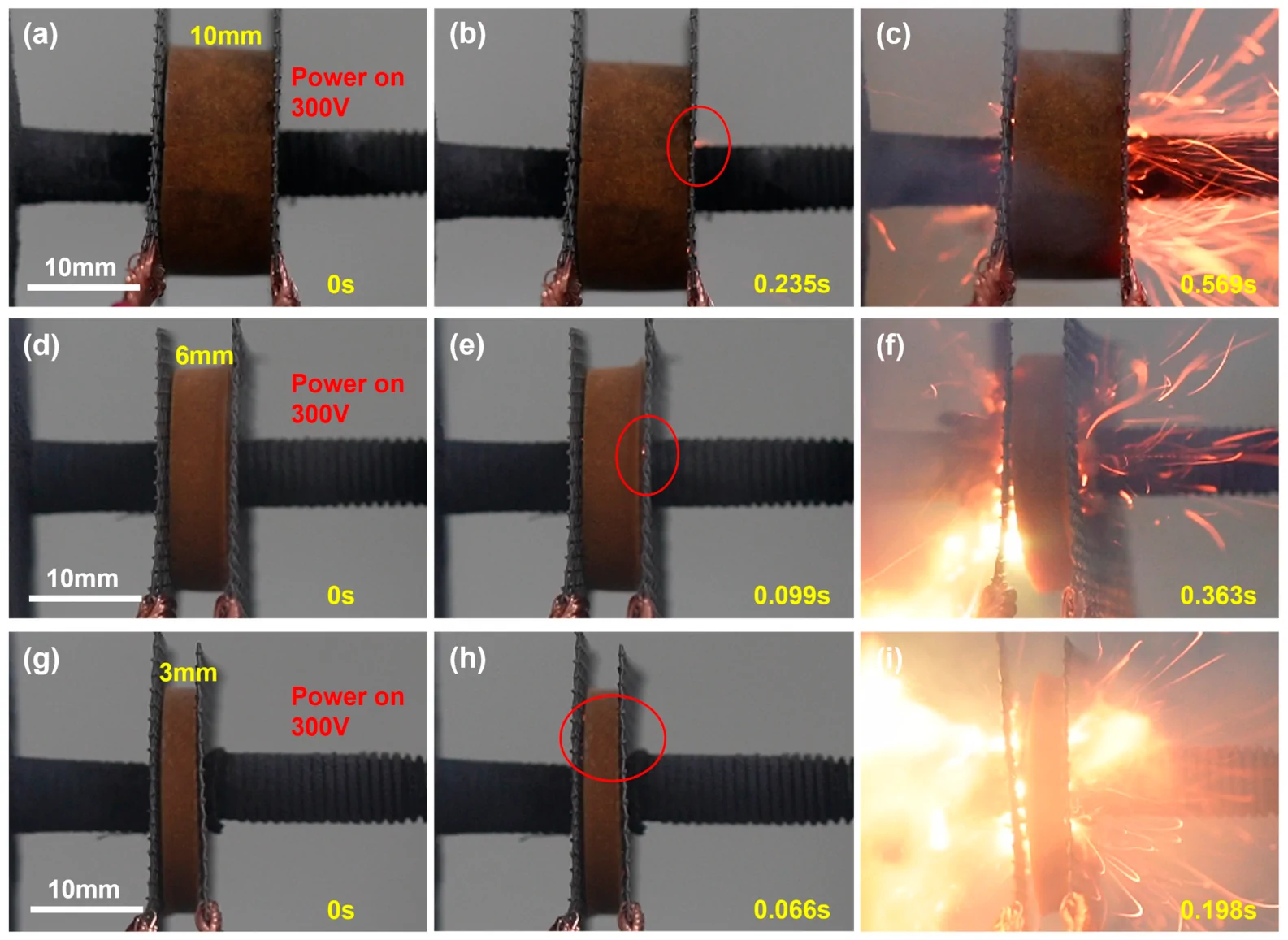
Ignition process of ECSP with different thicknesses (300 V, Ti mesh electrodes, wire diameter 0.3 mm, 20 mesh). (**a**–**c**) ECSP thickness 10 mm: appearance of cathode spark, formation of molten zone, and flame propagation toward the anode are shown sequentially; (**d**–**f**) ECSP thickness 6 mm; (**g**–**i**) ECSP thickness 3 mm. The position marked with a red circle is the initial ignition point.

To examine the effect of ECSP thickness on combustion performance, ignition tests were performed on cylindrical ECSP specimens with a diameter of 20 mm and thicknesses of 10 mm, 6 mm, and 3 mm. Ti mesh electrodes with a wire diameter of 0.3 mm and mesh number of 20 were used, and a DC voltage of 300 V was applied. The left electrode was connected to the positive terminal and the right electrode to the negative terminal, as shown in Figure 8.

The experimental results show that ignition was consistently first initiated at the cathode (right side), suggesting a cathode-dominated ignition mechanism. In addition, the ignition delay time increased with increasing ECSP thickness. Each experiment was repeated three times, and the ignition images from the repeated experiments are shown in Figure S4. For ECSP thicknesses of 10, 6, and 3 mm, the ignition delay times were 0.345, 0.09733, and 0.057 s, respectively, with standard deviations of 0.10082, 0.00153, and 0.01646 s, respectively.

This trend is attributed to the fact that thicker ECSP samples result in higher overall circuit resistance, which impedes electrochemical reactions and thus prolongs the reaction time. Even for thicker ECSP samples, the ignition process follows the general mechanism: spark first appears at the cathode, followed by the formation of a molten zone, and as the reaction propagates toward the anode, a flame emerges at the anode, as shown in Figure 8a–c. However, when the ECSP thickness was less than 3 mm, excessive voltage tended to cause simultaneous combustion at both the cathode and anode, resulting in uncontrolled combustion, as shown in Figure 8d–i. According to Hu et al., at a gap distance of 4 mm, high-temperature flames can readily induce breakdown discharge under high voltage, leading to unstable ECSP combustion. The breakdown thickness also increases with increasing applied voltage.

### 3.6. Effect of Applied Voltage on ECSP Combustion Performance

To investigate the effect of applied voltage on ECSP combustion performance, ignition tests were conducted on cylindrical ECSP specimens with a thickness of 10 mm and a cross-sectional diameter of 20 mm. Ti mesh electrodes (wire diameter 0.3 mm, 20 mesh) were used, and different DC voltage levels were applied. In the setup, the left electrode was connected to the positive terminal and the right electrode to the negative terminal.

**Figure 9:**
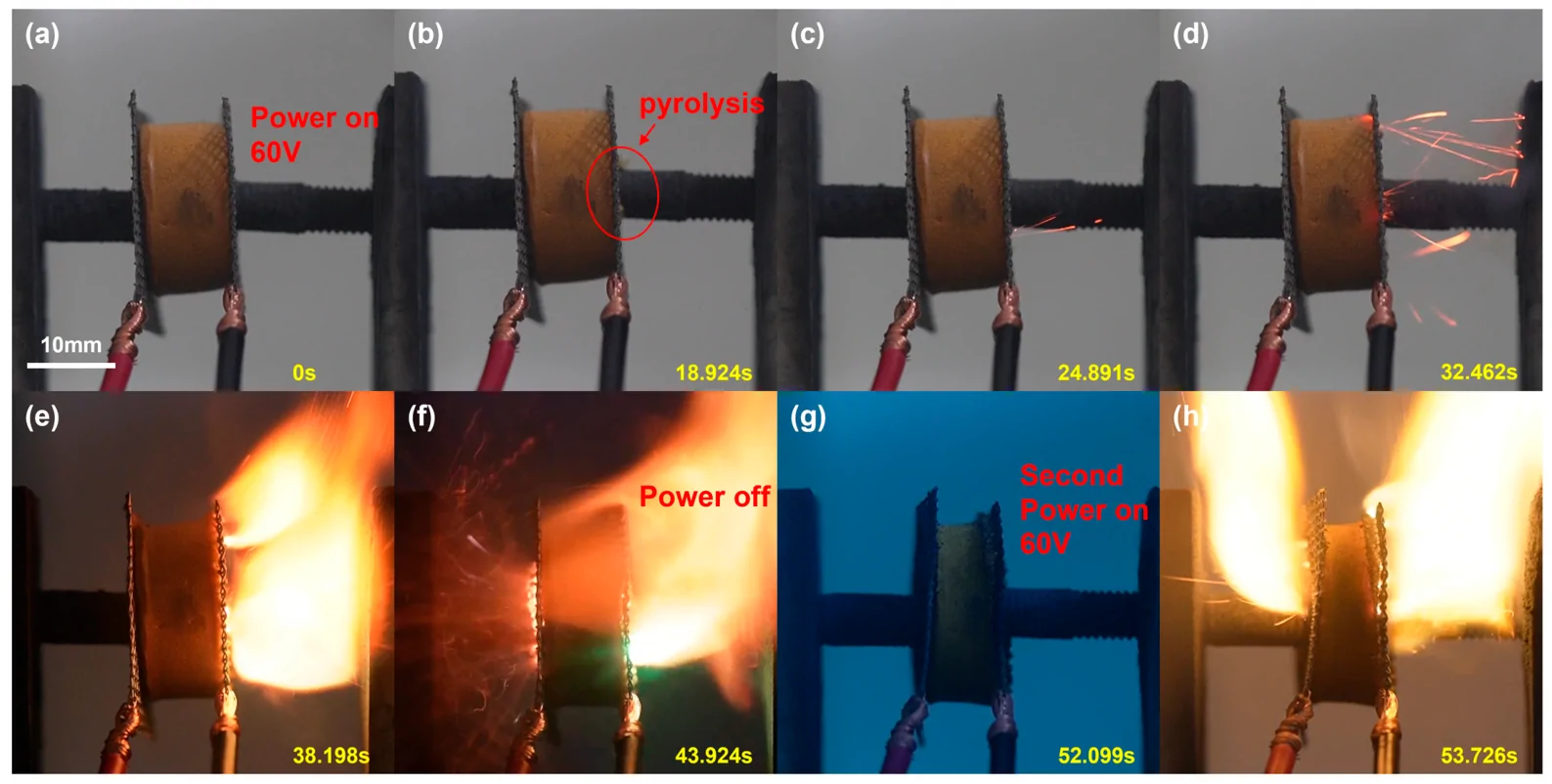
Ignition, extinguishment, and re-ignition process of ECSP at 60 V (Ti mesh electrodes, wire diameter 0.3 mm, 20 mesh). (**a**) 0 s (power-on instant); (**b**) 18.924 s (thermal decomposition produces bubbles, providing ionic channels); (**c**) 24.891 s (ignition energy threshold reached, ignition occurs); (**d**) 32.462 s (flame continues to develop); (**e**) 38.198 s (full combustion); (**f**) 43.924 s (power off, flame extinguishes); (**g**) 52.099 s (power reapplied); (**h**) 53.726 s (successful re-ignition, continuing the reaction process initiated during the first ignition). The position marked with a red circle is the initial ignition point.

Under the present electrode configuration and test method, ignition at 60 V was difficult and required a prolonged electrolysis process prior to ignition. Taking the instant of power application as zero time, as shown in Figure 9a, the electrochemical reactions inside the ECSP were difficult to initiate at this voltage. The temperature rise caused by Joule heating induced thermal decomposition of the propellant, generating gas bubbles (Figure 9b). This thermal decomposition provided ionic pathways for the electrochemical reactions, thereby promoting their progression. As electrolysis proceeded, the current through the ECSP gradually increased. Thermal decomposition and electrode reactions continued for 24.891 s. Once the ignition energy threshold was reached, the ECSP ignited (Figure 9c–e). At 38.198 s, the ECSP was fully ignited. Power was cut off at 43.924 s (Figure 9f), and the flame subsequently extinguished. Power was reapplied at 52.099 s (Figure 9g). Upon the second power application, the ignition process resumed from the stage established during the first ignition, showing a rapid response, with the ECSP re-ignited at 53.726 s (Figure 9h).

**Figure 10:**
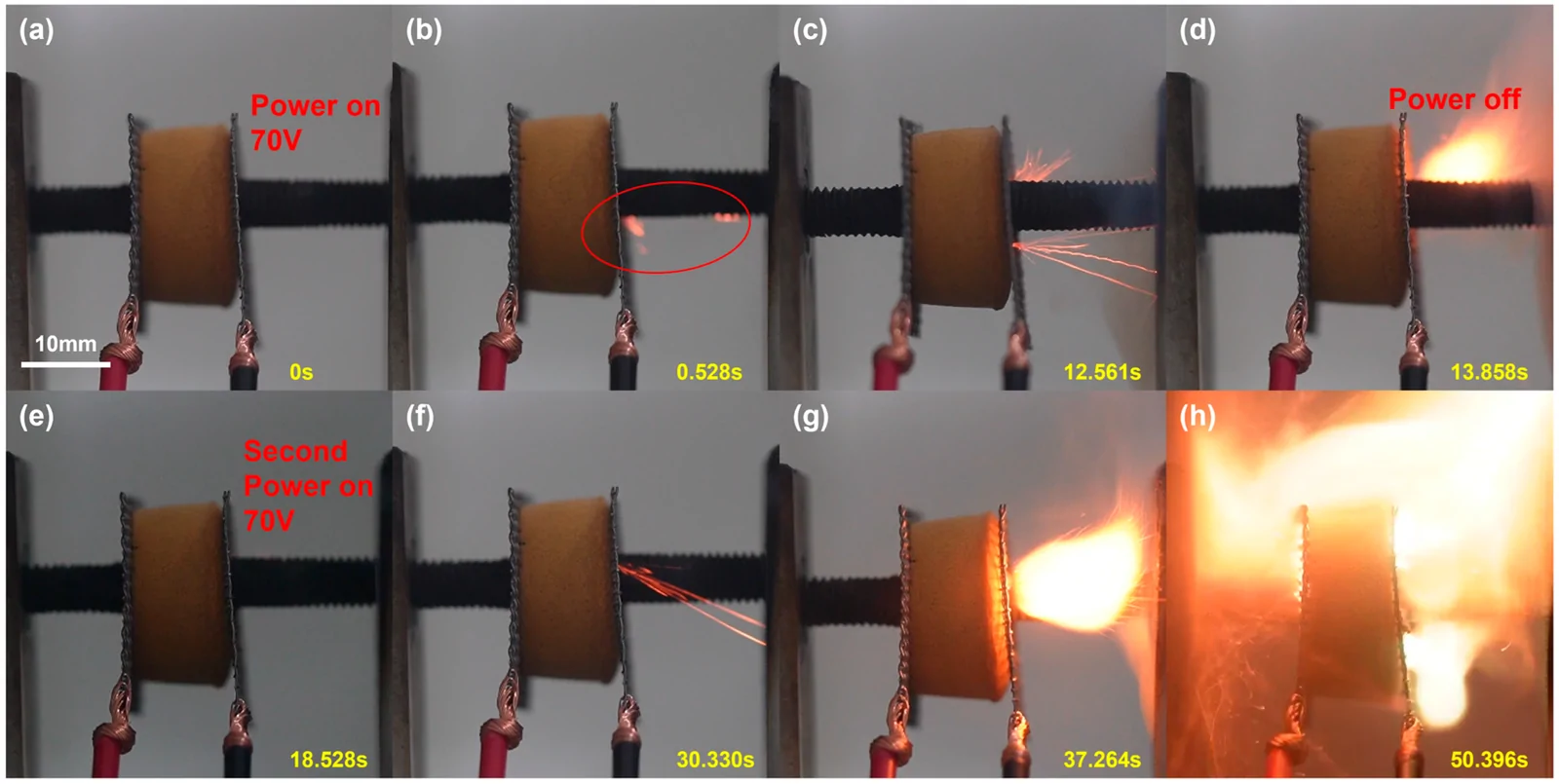
Ignition, extinguishment, and re-ignition process of ECSP at 70 V (Ti mesh electrodes, wire diameter 0.3 mm, 20 mesh). (**a**) 0 s (power-on instant); (**b**) 0.528 s (rapid ignition of ECSP); (**c**) 12.561 s (flame continues to burn stably); (**d**) 13.858 s (power off, flame gradually weakens and extinguishes); (**e**) 18.528 s (flame completely extinguished, power reapplied); (**f**) 30.330 s (flame rapidly re-established, electrolysis continues); (**g**) 37.264 s (sustained local combustion); (**h**) 50.396 s (full ignition achieved). The position marked with a red circle is the initial ignition point.

Under the present electrode configuration and test method, ignition at 70 V was considerably faster than that at 60 V. Taking the instant of power application as zero time (Figure 10a), the ECSP ignited at 0.528 s as the electrochemical reactions proceeded (Figure 10b). These results indicate that, under this electrode configuration and test method, ignition was difficult to achieve at voltages below 60 V but was readily achieved at voltages above 70 V. When power was cut off at 13.858 s, the ECSP extinguished (Figure 10d,e). Comparison of the two re-ignition processes shows that once the ECSP had been fully ignited, re-ignition after flame extinction occurred very rapidly. In contrast, when the initial ignition was incomplete, an additional period of electrolysis was required after extinguishment to achieve full re-ignition (Figure 10e,f). Re-ignition continues the process initiated during the first ignition, and the completeness of the first ignition also affects the re-ignition delay time.

The ignition behavior at different voltages indicates that, under the present electrode configuration and test method, ignition was difficult to achieve at applied voltages below 60 V because the Joule heat generated was insufficient to fully pyrolyze the ECSP, thereby preventing the formation of the ionic channels required for electrolysis. In contrast, ignition was readily achieved at voltages above 70 V, although the pyrolysis and electrolysis rates were substantially lower than those at 300 V. This suggests that, under these conditions, voltage influences the ignition delay time of ECSP by modulating the rates of the pyrolysis and electrolysis reactions.

To further verify this conclusion, experiments were conducted at 55, 60, 65, and 70 V, and the results are shown in Figure S5. At 55 V, the ECSP underwent prolonged electrolysis and pyrolysis prior to ignition. When the voltage was increased to 60 V, the duration of this process decreased. At 70 V, the electrolysis and pyrolysis reactions proceeded rapidly, and the ECSP ignited rapidly. The experiments were repeated twice, yielding consistent results.

## 4. Discussion

The measured ignition voltage and ignition delay are material-level characteristics. Without accounting for propellant grain geometry, propellant mass, electrode configuration, chamber pressure, initial temperature, and power-supply constraints, they cannot be directly translated into motor-level requirements. This issue has also been identified as a limitation and as a topic for future work.

## 5. Conclusions

Flame-retardant fillers significantly modulate ECSP resistivity: phenolic resin increases resistivity by 82.8% (to 78.33 Ω·m), while decabromodiphenyl ether decreases it by 32.3% (to 29.04 Ω·m), compared with the base value of 42.9 Ω·m.

Current density concentrates at electrode–propellant contact points and decreases with increasing wire diameter and mesh number; average current density exhibits a non-monotonic trend with mesh number, suggesting an optimal range for uniform current distribution.

Pore size exerts a stronger influence on interfacial thermal stress than wire diameter; larger pores increase local stress and deformation, raising the risk of debonding. A 0.5 mm wire diameter with 2 mm pore size offers a favorable balance.

In asymmetric electrode configurations (8-mesh anode vs. 10-mesh cathode), current density disparity dominates the ignition sequence over polarity effects.

Electrode material affects ignition priority in the order Ti > SS > Cu, independent of polarity, indicating that material properties play a critical role in determining preferential ignition sites.

Ignition delay increases with increasing propellant thickness. At thicknesses below 3 mm, high voltage readily induces uncontrolled combustion.

Under the present electrode configuration and test method, ignition was difficult below 60 V but readily achieved above 70 V; below 60 V, insufficient Joule heating prevented formation of the ionic channels required for electrolysis. Re-ignition delay depended on the completeness of the initial ignition.

## Figures and Tables

**Table 1:** Material property definition for finite element analysis.

	Electrical Resistivity (Ω·m)	Thermal Conductivity (W·m^−1^·K^−1^)	Density (kg·m^−3^)	Young’s Modulus (Pa)	Poisson’s Ratio
Ti	4.8 × 10^−7^	16.4	4510	1.1 × 10^11^	0.34
ECSP	42.9	0.35	1420	5 × 10^6^	0.42

The electrical resistivity of ECSP was determined experimentally, whereas the remaining material parameters were adopted from literature values [14].

## Data Availability

The original contributions presented in this study are included in the article/Supplementary Materials. Further inquiries can be directed to the corresponding authors.

## Supplementary Materials

The following supporting information can be downloaded at: https://www.mdpi.com/article/10.3390/ma19204297/s1, Figure S1. Schematic illustration of the ECSP sample preparation process. Figure S2. Axial displacement of the ECSP end face in contact with mesh electrodes of different aperture sizes. Figure S3. Axial displacement of the ECSP end face in contact with mesh electrodes of different wire diameters. Figure S4. Images captured at the ignition moment for ECSP samples of different thicknesses (300 V; Ti mesh electrode; wire diameter, 0.3 mm; 20 mesh). Figure S5. Images captured at the ignition moment for ECSP at applied voltages of 55, 60, 65, and 70 V (propellant thickness, 10 mm; Ti mesh electrode; wire diameter, 0.3 mm; 20 mesh).
