# Supplementary material for: Effects of Formulation and Electrode Parameters on the Ignition Performance of Electrically Controlled Solid Propellant
Source: Materials (Basel). 2026 Oct 12;19(20):4297. doi: 10.3390/ma19204297 (PMC13706380; doi:10.3390/ma19204297)
Supplement: Supplementary file 1 [file materials-19-04297-s001.zip › materials-4584556-supplementary.pdf]

Supplementary Materials

Effects of Formulation and Electrode Parameters on the Ignition Performance of Electrically Controlled Solid Propellant

Authors: Rongyao Zhi, Zhe Zhang, Xiong Zhou, Yanan Wang, Gang Chen, Guodong Li*, Yin Huang*.
Corresponding author: guodonglee@whut.edu.cn, huangyin@casc42.cn;

This file includes:

Figures S1 to S5

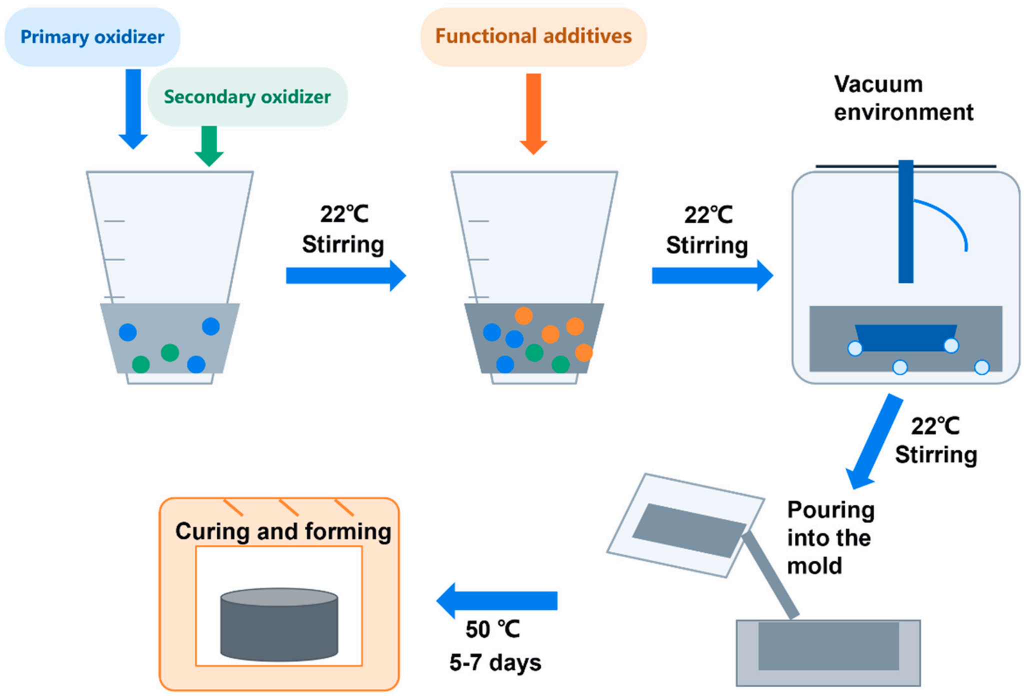

Figure. S1. Schematic illustration of the ECSP sample preparation process.

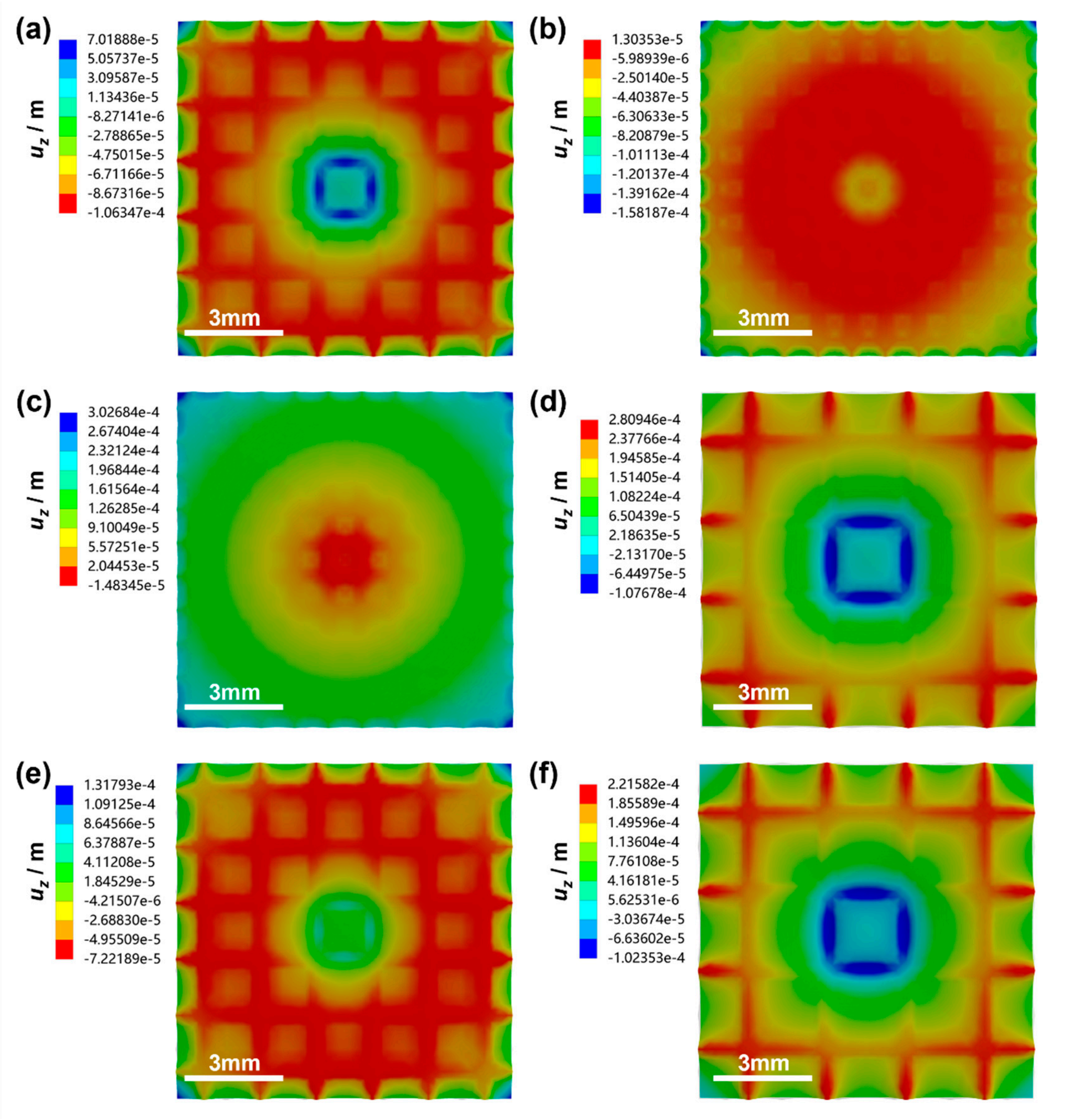

Figure. S2. Axial displacement of the ECSP end face in contact with mesh electrodes of different aperture sizes. (a) and (b) correspond to the axial displacement of ECSP at the contact interfaces with mesh electrodes having a wire diameter of 0.5 mm and aperture sizes of 2 and 1, respectively, as shown in Fig. 6(a); (c) and (d) correspond to the axial displacement of ECSP at the contact interfaces with mesh electrodes having a wire diameter of 0.5 mm and aperture sizes of 1 and 3, respectively, as shown in Fig. 6(b); (e) and (f) correspond to the axial displacement of ECSP at the contact interfaces with mesh electrodes having a wire diameter of 0.5 mm and aperture sizes of 2 and 3, respectively, as shown in Fig. 6(c).

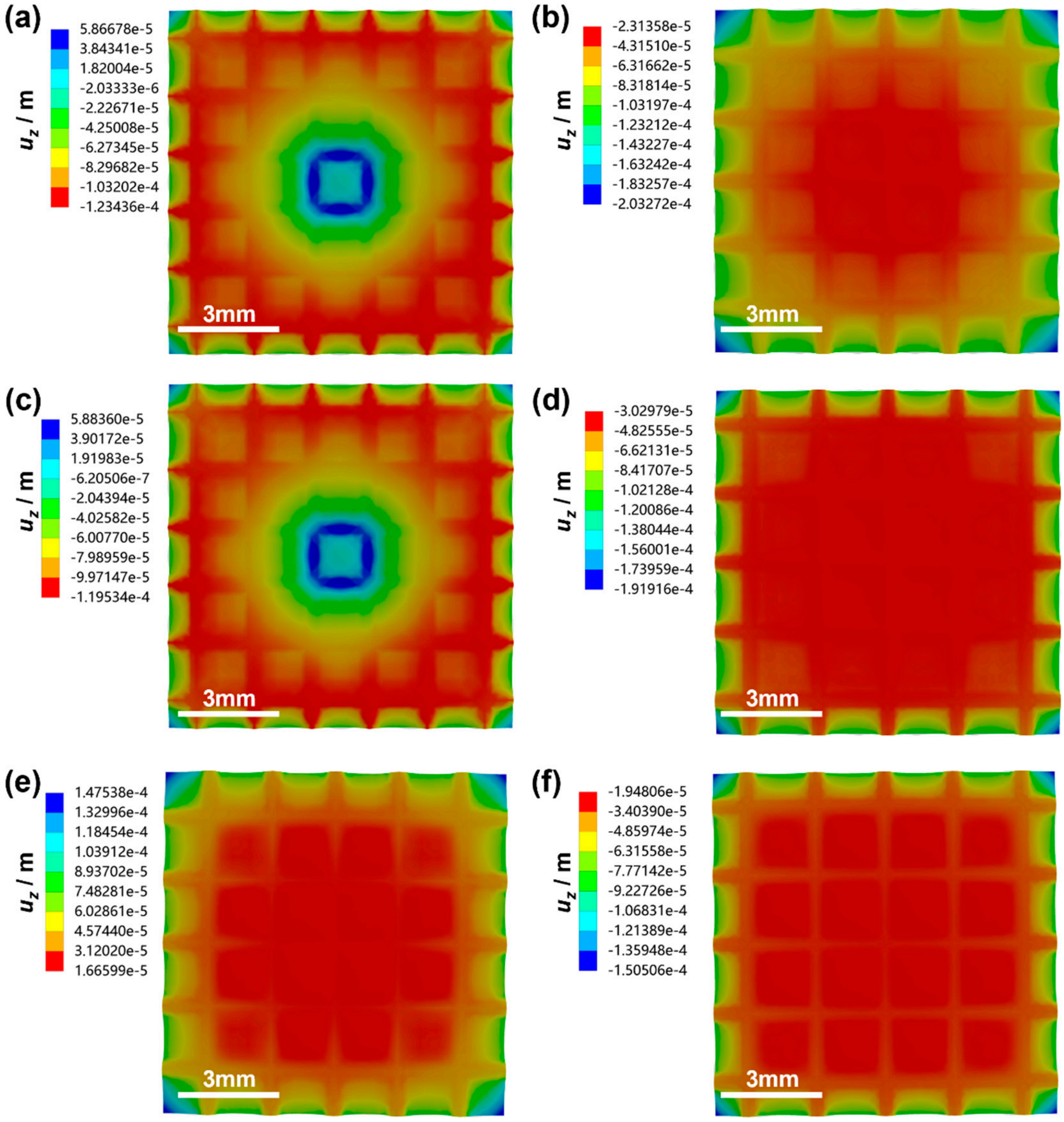

Figure. S3. Axial displacement of the ECSP end face in contact with mesh electrodes of different wire diameters. (a) and (b) correspond to the axial displacement of ECSP at the contact interfaces with mesh electrodes having wire diameters of 0.5 and 0.75 mm and an aperture size of 2, respectively, as shown in Fig. 6(d); (c) and (d) correspond to the axial displacement of ECSP at the contact interfaces with mesh electrodes having wire diameters of 0.5 and 1 mm and an aperture size of 2, respectively, as shown in Fig. 6(e); (e) and (f) correspond to the axial displacement of ECSP at the contact interfaces with mesh electrodes having wire diameters of 0.75 and 1 mm and an aperture size of 2, respectively, as shown in Fig. 6(f).

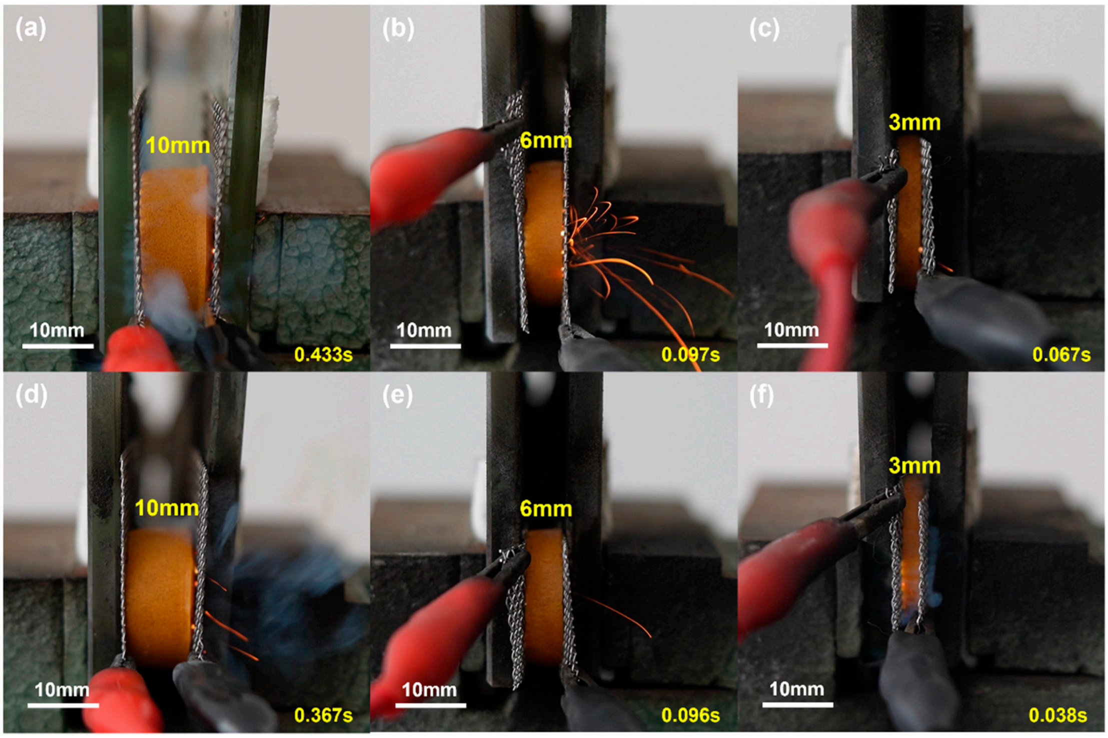

Figure. S4. Images captured at the ignition moment for ECSP samples of different thicknesses (300 V; Ti mesh electrode; wire diameter, 0.3 mm; 20 mesh). (a) and (d) ECSP thickness, 10 mm; ignition delays, 0.433 and 0.367 s. (b) and (e) ECSP thickness, 6 mm; ignition delays, 0.097 and 0.096 s. (c) and (f) ECSP thickness, 3 mm; ignition delays, 0.067 and 0.038 s.

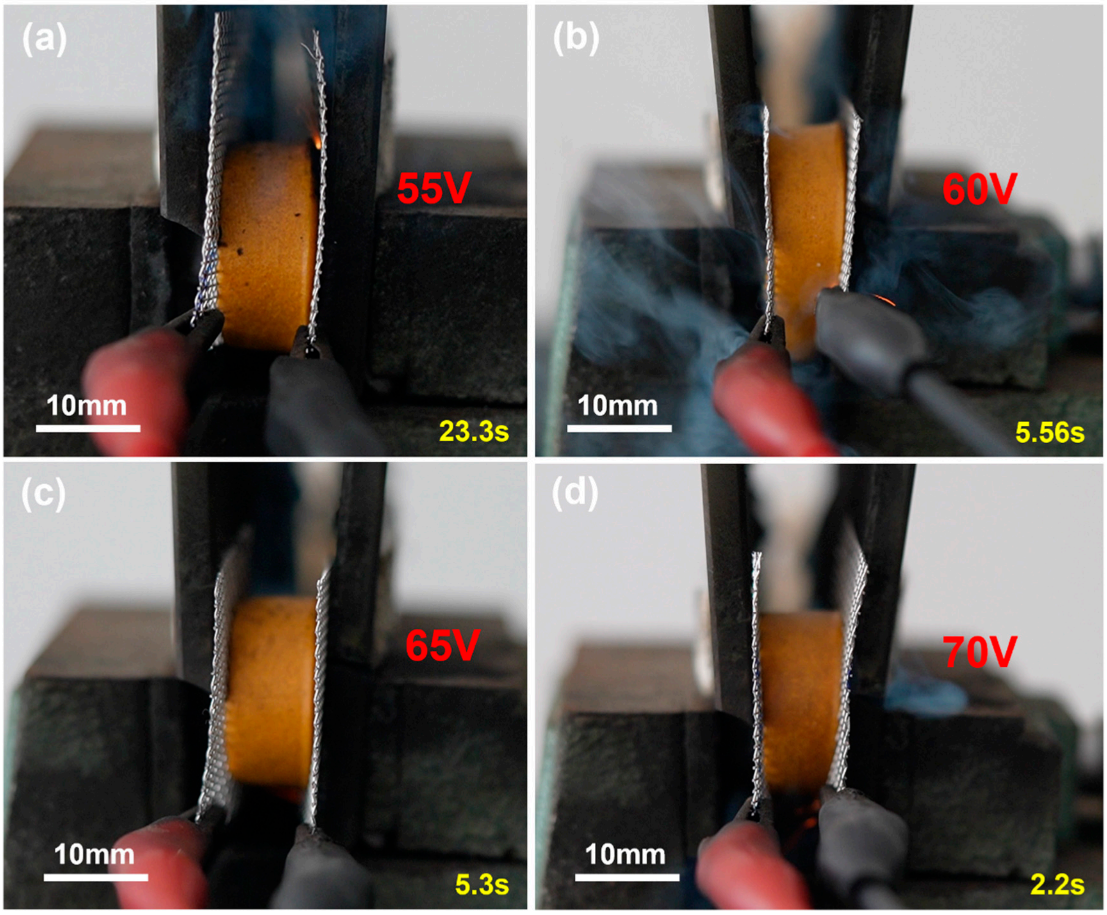

Figure. S5. Images captured at the ignition moment for ECSP at applied voltages of 55, 60, 65, and 70 V (propellant thickness, 10 mm; Ti mesh electrode; wire diameter, 0.3 mm; 20 mesh). (a) At 55 V, ECSP underwent prolonged electrolysis and pyrolysis before ignition. (b) At 60 V, the duration of this process decreased. (c) At 65 V, the duration decreased further. (d) At 70 V, the electrolysis and pyrolysis reactions proceeded rapidly, enabling rapid ignition of ECSP. Each experiment was repeated twice, yielding consistent results.
